# Annual Meeting of the American Medical Association

**Published:** 1868-05

**Authors:** 


### Miscellaneous.

### Annual Meeting of the American Medical Association.

The nineteenth annual session of the American Medical Association was held in Washington, commencing Tuesday, 6th inst. The Convention was called to order by the President, Dr. L. D. Gross of Philadelphia, who introduced Dr. Grafton Tyler of Georgetown, who delivered an address of welcome, after which Professor Gross delivered his inaugural address. He returned his profound gratitude for the distinguished honor conferred upon him in placing him in a position so often occupied by the distinguished men of the profession. For this mark of respect and confidence he could only promise to do his duty impartially, and, as far as possible, expedite the business of the Association. The Professor then, at length, explained the object and ends of the Association, the duties of the profession, the advancement of the science, the brilliancy of the rank attained by the American faculty in the ranks of the profession throughout the world, the great good the Association has done in the past twenty years by its annual gatherings, and the continued good promised by its uninterrupted meetings. He gave at length his views on the reception of prize essays on medical subjects, and of the duties of professors of colleges, the management of hospitals, etc. He spoke in eloquent and impressive language of the departed members of the Association, and was exceedingly brilliant in his hopes and sanguine expectations of a bright future for this great Republic; entreated the great fraternity to go on doing good, as usual, throughout the length and breadth of the land, and concluded by again returning thanks to the Association for the honor they have conferred upon him.

At the conclusion of the address, on motion, it was ordered to be printed.

The regular business next followed—and the committees were called. Most of the committees responded as they were called, and their reports were referred to their appropriate sections.

The Committee on Medical Ethics, on consultation with female practitioners, made a report, closing with the following resolution:

*Resolved*, That the question of sex has never been considered by this Association in connection with consultations among medical practitioners, and that in the opinion of this meeting, every member of this body has a perfect right to consult with any one who presents the "only presumptive evidence of professional abilities and requirements" required by this Association, viz: "a regular medical education."

The Committee on Prize Essays reported that four essays had been submitted, and the two prizes of $100 each were still in the hands of the committee, as no award had been made. They recommended that both should be offered for the best essay, but the subject was indefinitely postponed.

SECOND DAY.

A number of papers were received and referred to the various committees.

The Association took a recess of fifteen minutes in order to give the various State delegations an opportunity to select a member from their respective States to form the nominating committee for the ensuing year.

At 10½ o'clock the Association re-assembled, and the names of the delegates selected were sent in. They are as follows:

Maine, Dr. N. P. Monroe; New Hampshire, Dr. C. B. Twitchell; Massachusetts, Dr. H. R. Storer; Rhode Island, Dr. O. Bullock; Connecticut, Dr. A. Woodward; New York, Dr. Arnesby; New Jersey, Dr. S. Lilley; Pennsylvania, Dr. S. Pollock; Delaware, Dr. H. Askew; Maryland, Dr. T. J. Hellsby; Virginia, Dr. Owen; West Virginia, Dr. Cummings; Georgia, Dr. R. D. Arnold; Ohio, Dr. W. H. Mussey; Illinois, Dr. Hildreth; Tennessee, Dr. Kellar; Alabama, Dr. Wetherly; Indiana, Dr. Sutton; Iowa, Dr. Clever; Michigan, Dr. Palmer, District of Columbia, Dr. F. Howard; U. S. Army, Dr. G. A. Otis.

Prof. John Gange, veterinary surgeon from Prince Albert College, London, was introduced to the Convention, and delivered a very interesting address. Senator Drake, of Missouri, who wae present, was invited to the stand, and he delivered an interesting address.

The report of the Committee on Medical Ethics was submitted. A lengthened debate then ensued in relation to the admission to practice of female physicians. Dr. Davis, of Chicago, moved that the question be indefinitely postponed, which motion was carried unanimously.

The resolution of acceptance of the resignation of Dr. Homberger was next taken up. Dr. Sayre moved that the name of Dr.

Homberger be stricken from the rolls because he had violated the code of ethics. After a lengthy debate as to whether the resignation should be accepted or the member expelled, the question was finally taken, and Dr. Homberger was expelled.

Dr. Hartman, of Baltimore, offered a resolution censuring those physicians of Baltimore who endorsed a certain foreign specialist by permitting the use of their names in his behalf. Referred to the Committee on Ethics.

The Convention then adjourned to meet at 9 o'clock on Thursday morning.

The Medical Association united in a body on Wednesday night, at the United States Medical Museum on Tenth street, where Dr. Woodward, of that institution, delineated the microscopical views of the intestines as affected by dysentery, and the eruption of the skin as caused by camp-fever and small-pox, which views were, in many instances, thrown upon a screen or piece of canvass twelve feet square and magnified to nearly that extent. This exhibition was very fine and received the admiration of the members present. The entertainment at the Army Medical Museum was one of the greatest attractions of the session.

The medical fraternity proceeded from the Museum to the residence of Senator Morgan, corner of Fifteenth and I streets, where they were received in fine style by the distinguished Senator and the accomplished ladies of his household.

THIRD DAY.

The Convention resumed its session, Dr. Gross in the chair.

The report of the Committee on Nominations was presented and accepted. The report names New Orleans, La., as the place to hold the next meeting of the Convention, and fixes the time for May next.

The following officers of the Convention were nominated by the committee:

President, Dr. William O. Baldwin, of Alabama; first Vice President, Dr. George Mendenhall, of Ohio; second Vice President, Dr. Noble Young, of Washington, D. C.; third Vice President, Dr. N. P. Monroe, of Maine; fourth Vice President, Dr. S. M. Bemis, of Louisiana; Treasurer, Dr. Caspar Wistar, of Philadelphia; Committee on Publication, Dr. Francis G. Smith, jr., of Philadelphia, chairman; Dr. Wm. B. Atkinson, of Philadelphia;

Dr. H. F. Askew, of Delaware; Dr. Richard M. Cooper, of New Jersey; Dr. J. H. Lovejoy, of the District of Columbia; Dr. Wm. Marbury, of Pennsylvania.

The Committee on President's Address made their report, accompanied by a series of resolutions in relation to the appointment of various committees, five of which were adopted, and the sixth was referred to a special committee. The Chair appointed the following committees:

Commissioners to aid in trials involving Scientific Testimony—Drs. John Ordenaux, of New York; A. B. Palmer, of Michigan; Stephen Smith, of New York; J. W. Dunbar, of Baltimore.

Annual Medical Register—Drs. Packard of Philadelphia, Wm. B. Bibbins of New York, and Ellsworth Elliott of New York.

Devising a Plan for the Relief of Widows and Orphans of Medical Men—Drs. J. H. Griscam of New York, N. S. Davis of Illinois, and A. C. Post of New York.

Veterinary College—Drs. Thomas Antisell of Washington, D. C., C. C. Lee of New York, and John C. Dalton of New York.

The Chair appointed the following delegates to represent the American Medical Association in Canada, to meet in September next:—C. C. Cox, M. D., LL. D., of Maryland; Drs. John Atlee, of Pennsylvania; N. S. Davis, of Illinois; Charles C. Lee, of New York, Grafton Tyler, of D. C.; ——— Wood, of the Navy, and S. D. Gross.

Dr. C. C. Cox of Maryland, read the report on American Medical Necrology, which was ordered to be printed.

Dr. Baldwin of Alabama, the newly elected President of the Association for the current year, was introduced, and returned his thanks in appropriate terms. At the close of Dr. Baldwin's remarks, Dr. Gross, the retiring President, arose and said that he desired to correct a misapprehension which existed at the South, that the Medical Association during the war had requested Congress to enact a law making all medicines and surgical instruments contraband of war. No such resolution had ever been introduced.

An invitation was read from the Young Men's Christian Association of this city for the Association to visit their library and reading rooms.

Dr. N. S. Davis of Illinois, offered a resolution instructing the Chair to appoint a committee of three to report at the next session on the practicability of establishing a library of American medical works, including books, monograms and periodicals.—Adopted.

About 8 o'clock in the evening the Association visited the Capitol, which had been illuminated for the occasion. Some time was spent in examining the electrical apparatus, the workings of which were explained by Prof. Gardner. The distinguished party then proceeded to Chief Justice Chase's residence. Dr. D. W. Johnson of this city, and Dr. Grafton Tyler of Georgetown, introduced the party, who, after an exchange of courtesies, sat down to a bounteous supper. From Justice Chase's the Association visited Mayor Wallach, where they were cordially received, and enjoyed themselves in social converse, concluding with a fine collation.

This Association concluded its fourth and last day's deliberation on Friday in signal harmony and good feeling. Representing nearly all the States, and including the most distinguished members of the profession, no body ever assembled in the National Capital entitled to more respect or wielding a larger influence in and out of the circle of their labors. Their sessions were attended by choice audiences, and they were the recipients of many elegant hospitalities. The President of the United States, Chief Justice Chase, Speaker Colfax, Mayor Wallach, Hon. E. D. Morgan, and others, entertained them with refined and considerate generosity. The annual meetings of this Association, numerously attended before the war, were confined almost exclusively to the adhering States, during hostilities, and ever since the overthrow of the rebellion, various reasons, political and personal, have conspired to prevent the general harmony of other and happier days. The Convention which adjourned on Friday, however, was essentially larger than on any occasion since 1860.—*Abstracted from the Daily Washington Chronicle.*

